# Muscle wasting in icu trauma patients: quantitative and qualitative changes

**DOI:** 10.1186/2197-425X-3-S1-A181

**Published:** 2015-10-01

**Authors:** MG Annetta, D Silvestri, DL Grieco, MF La Torre, N Magarelli, C Policola, A Caricato, S Della Casa, M Antonelli

**Affiliations:** Catholic University of Rome, Anesthesiology and Intensive Care Medicine, Rome, Italy; Diagnostic Radiology, Catholic University of Rome, Rome, Italy; Catholic University of Rome, Endocrinology, Rome Italy

## Introduction

Loss of skeletal muscle is a typical and early finding in critically ill patients, mostly related to forced immobility, systemic inflammatory response, and protein depletion; it is clinically associated with a functional impairment that may cause difficult weaning from mechanical ventilation, increased length of ICU and hospital stay and long term functional disability.

## Objectives

We carried out a prospective observational study to evaluate the morphological changes of the muscle mass in a group of enterally fed trauma patients, comparing them with a control group of malnourished patients (anorexia nervosa).

## Methods

Fifteen ICU patients were studied on day 1 and on day 20 after trauma and compared with 15 anorexic patients with severe malnutrition (BMI < 17) volunteering to participate to the study. Muscle mass was evaluated by ultrasound scan, a recently validated method with several advantages: it is accurate, non-invasive, inexpensive, easily repeatable, easy to perform at bedside and apt to detect both qualitative and quantitative changes of the muscle. In all patients, we studied the rectus femuris (RF) and anterior tibialis (AT) muscles, considering

(a) the anterior-posterior diameter,

(b) the lateral diameter,

(c) the sectional area and

(d) the muscle echogenicity according to Heckmatt's score.

## Results

Our trauma patients were 12 males and 3 females; age was 53 (range 43-61), length of ICU stay 35 days (23-50), days on mechanical ventilation 22 (12-36), injury severity score 34 (27-50), BMI 27.5 (23.1-29.4). The anorexic patients were all female: age 21.5 (17-30), BMI 14.9 (13.2-16.65). From day 1 to day 20 after trauma, the muscle mass decreased significantly, reaching the values observed in the anorexic patients: the anterior-posterior diameter of both RF and AT decreased significantly (p < 0.008 and p < 0.001, respectively); the area of RF decreased by 26.1% (16.1 % - 36.3%, p < 0.001); the area of AT decreased by 37.3% (24.5% - 50.2%, p < 0.001), reaching a value not dissimilar from anorexic patients (p = 0.18). On day 1 after trauma, muscle echogenicity of both RF and TA was higher than in anorexic patients (p < 0.001), suggesting very early inflammatory changes. The muscle echogenicity was higher on day 20 if compared to day 1, both in RF (p < 0.005) and in AT (p < 0.004), suggesting further abnormalities of muscle structure which cannot be explained by malnutrition alone.

## Conclusions

Evaluation of skeletal muscle changes in the critically ill patients can be successfully performed by ultrasound, which yields both quantitative and qualitative data. Our results confirm the progressive depletion of muscle mass in the ICU trauma patient; also, our data suggest that the inflammatory process - which is a major contributor of muscle wasting - begins very early in the critical illness, in the first 24 hrs after trauma, and progress further during the ICU stay.

